# Occupational therapists’ role in sleep management in palliative care: A cross-sectional survey

**DOI:** 10.1177/03080226251352648

**Published:** 2025-07-21

**Authors:** Madeleine Webster, Linda Barclay, Dhwani Parikh, Aislinn Lalor

**Affiliations:** 1 Arliam Allied Health, MOIRA, Scoresby, VIC, Australia; 2 Department of Occupational Therapy, Monash University, Melbourne, VIC, Australia; 3 Palliative Care South East, Cranbourne, VIC, Australia; 4 Rehabilitation, Ageing and Independent Living Research Centre, Monash University, Melbourne, VIC, Australia

**Keywords:** Palliative care, sleep, end of life, sleep management

## Abstract

**Introduction:**

Sleep is fundamental to an individual’s health, well-being and quality of life. Poor sleep and sleep disturbances are common for individuals receiving palliative care. Occupational therapists play a crucial role by effectively addressing sleep in this context. However, there has been limited research regarding the role, scope and implementation of sleep management interventions among occupational therapists within palliative care, and the needs that therapists have to support this area of practice.

**Methods:**

An online cross-sectional survey was conducted among Australian occupational therapists with current or recent experience in palliative care. Qualitative data from open-ended response items were analysed using content analysis.

**Results:**

Fifty-one occupational therapists (92.2% female) with an average of 7.6 years of experience working in palliative care completed the online survey. Most participants (88.2%) perceived that sleep was within the scope of the occupational therapy practice in palliative care; however, few participants indicated good/excellent knowledge of sleep or sleep assessment and intervention. Barriers to adequately addressing sleep issues included a lack of therapists’ knowledge, limited understanding of the scope by other professionals, workload constraints and limited resources.

**Conclusion:**

Occupational therapists may benefit from evidence-based resources and guidelines to address sleep issues in palliative care.

## Introduction

Sleep is a complex phenomenon, fundamental for well-being and quality of life (Davies, 2019). Sleep plays a vital role in physiological and psychological functioning, having a profound influence on mood, energy, executive function and behaviour (Tester and Foss, 2018). Sleep disturbances refer to a variety of heterogeneous issues, including difficulty initiating or maintaining sleep, in addition to perceived or actual disturbances to sleep–wake cycles or sleep quality (Delgado-Guay et al., 2011).

Palliative care is person and family-centred care provided for a person with an active, progressive, advanced disease, who has little or no prospect of cure and who is expected to die, and for whom the primary goal is to optimise the quality of life (Palliative Care Australia, 2025). For this study, palliative care refers to care provided to adults by occupational therapists and other health professionals working in hospitals, residential aged care, hospices, generalist community services and specialist palliative care services. There is increasing demand for palliative care services in Australia, congruent with Australia’s ageing population and increased incidence of chronic, incurable disease (Australian Institute of Health and Welfare, 2021).

Sleep issues are commonly experienced by individuals receiving palliative care and their caregivers (Davies, 2019). Poor sleep is associated with increased symptom distress, reduced quality of life and increased healthcare costs in palliative care (Sagha Zadeh et al., 2018). Delgado-Guay et al. (2011) identified associations between sleep disturbances and increased frequency of pain, anxiety, depression and decreased well-being among advanced cancer patients receiving palliative care. Emerging evidence supports the use of nonpharmacological interventions to effectively address sleep disturbances and reduce symptom distress for clients in palliative care (Capezuti, 2021; Capezuti et al., 2018; Sagha Zadeh et al., 2018).

Occupational therapists have an important role in supporting quality of life, health and well-being, and in facilitating a good death for clients in palliative care (Eva and Morgan, 2018; Hammill et al., 2019; Yeh and McColl, 2019). Occupational therapists provide occupation-focused, evidence-based interventions that support end-of-life participation and quality of life (Eva and Morgan, 2018). According to a study that aimed to map the scope of occupational therapy palliative care interventions across Europe, common interventions included the following: prescription of assistive technology and environmental modifications; positioning to support comfort and posture; strategies to manage daily activities; fatigue and pain management; assessing and supporting valued occupations; pressure injury management and anxiety management (Eva and Morgan, 2018). Even though sleep is a well-recognised and important occupation (Occupational Therapy Practice Framework, 2020), it was not included in the survey questions, nor identified by participants in the free-text response options (Eva and Morgan, 2018). Occupational therapy is known to be misunderstood and underutilised in palliative care settings (Knecht-Sabres et al., 2019; Talbot-Coulombe et al., 2022), which could be one reason for the low uptake of sleep-based interventions.

Research has identified the important role of occupational therapy in improving sleep across various other practice settings, including within mental health (Faulkner and Mairs, 2015), with older adults (Leland et al., 2014), and with young children and their families (Gronski and Doherty, 2020). Sleep-related interventions identified include the following: addressing evening routines that incorporate desired sleep behaviours; sleep hygiene advice and education, including facilitating supportive environments and habits that promote restful and adequate sleep; and modifying daily routines to include appropriate activity participation. However, to date, there has been limited research conducted regarding the role, scope and implementation of sleep management interventions among occupational therapists within palliative care, and the needs that therapists have to support this area of practice. Therefore, this study aimed to investigate the role of Australian occupational therapists in sleep management with individuals receiving palliative care. The research questions for this study were as follows:

1. What are the roles of occupational therapists in Australia working in palliative care regarding sleep?
2. What do Australian occupational therapists working in palliative care perceive as facilitators and barriers to their role in sleep management in palliative care?
3. What current resources are used by, or would support Australian occupational therapists working in palliative care in their role in sleep management for people receiving palliative care?

## Methods

### Design and procedure

This study adopted an online cross-sectional survey design to examine the role of Australian occupational therapists working in palliative care. A cross-sectional survey design was chosen to provide a profile of the population at a single time point (Schofield and Forrester-Knauss, 2017). Online delivery was selected as it enables wide distribution, can be completed at a convenient time for participants and is cost-effective (Forsyth and Kviz, 2017). The reporting of this survey is guided by the CHERRIES (Checklist for Reporting Results of Internet E-Surveys) criteria (Eysenbach, 2012; Eysenback, 2004) by informing the necessary aspects that are required when reporting e-surveys. The survey was accessible to participants via Qualtrics^©^ (an online survey platform) for 10 weeks. Once ethics approval was obtained from Monash University, participants were provided with an explanatory statement at the start of the survey outlining the study’s purpose and details of the study, including ethical considerations of the research and participation requirements (including estimated completion time of 20–30 minutes). Participants were asked to indicate consent by completing a check box following the explanatory statement. No identifying information was obtained from participants to maintain their anonymity.

### Survey development

As there were no prior published surveys exploring this practice area, an online survey was developed by authors MW and AL to investigate the research aims, informed by (i) existing literature investigating occupational therapists’ experiences; (ii) drawing on current occupational therapy clinical expertise within palliative care and (iii) a broader survey conducted by Eva and Morgan (2018) in Europe. The survey consisted of 25 questions, including both open and closed response options, across 4 sections: (A) demographics (8 items); (B) occupational therapy role in sleep in palliative care (6 items); (C) resources (5 items), and (D) open response (6 items; refer to Supplemental Appendix A). In section B, participants rated their knowledge about sleep, sleep assessments and sleep interventions using a 5-point Likert scale ranging from ‘I have no knowledge’ to ‘Excellent – I know a lot’. In section C, participants were able to indicate whether a resource was useful, helpful and/or needed. Pretesting of the survey was completed prior to data collection with one occupational therapy student and a senior occupational therapist currently working in palliative care, who is also a subject area expert. This approach provided feedback to confirm that the survey elicited the desired information and facilitated the identification of any required changes (Schofield and Forrester-Knauss, 2017). The subject area expert occupational therapist was also able to provide comments to the survey developers (MW and AL) to assist with ensuring survey items adequately measured the concepts they intended to measure about palliative care. Following the feedback, no questions were removed or added; however, relevant changes and modifications to the survey formatting were made to ensure appropriate survey language, flow and clarity. Through this process, both face validity (e.g. survey items appear appropriate and relevant on the ‘face of it’) and content validity (e.g. items adequately measure concepts they intend to measure) (Latour and Tume, 2021) were obtained. As outlined by Forsyth and Kviz (2017), pretesting the survey with respondents with similar characteristics to potential participants ensures the clarity and relevance of survey questions.

### Participants

Convenience and snowball sampling methods were employed to enhance the number of potential participants reached (Parker et al., 2019). Participants were recruited via the research team’s social media (LinkedIn, Twitter and Facebook pages), occupational therapy networks including Occupational Therapy Australia’s online newsletter, Occupational Therapy Australia Palliative Care Special Interest Group email list and email to existing professional contacts. Potential participants who met the following inclusion criteria were invited to participate in the study: (i) be a qualified occupational therapist in Australia and (ii) be currently working in palliative care services with a minimum of 6 months of experience or have worked in palliative care services in Australia within the past 3 years.

A short advertisement, including a brief introduction to the study, an invitation to participate and a link to the explanatory statement and survey, was distributed to inform potential participants about study details and engage participant interest (Schofield and Forrester-Knauss, 2017). Potential participants were informed that participation in the research was voluntary and anonymous. To optimise the response rate, the study advertisement was reshared to social media and in follow-up emails approximately 2 weeks before the survey closed. The data collection period commenced on 18 March 2022 and concluded on 31 May 2022.

### Data analysis

Survey data were directly downloaded from Qualtrics into the IBM Statistical Package for Social Sciences (SPSS) version 27.0, IBM for management and analysis. Quantitative data obtained from close-ended survey items were analysed using descriptive statistics to determine frequencies and percentages (Forsyth and Kviz, 2017). Responses to items were allocated numerical value codes to ensure participant responses were appropriate for statistical analysis (e.g. Yes coded as 1, No as 0, or statements ranging with responses from ‘I have no knowledge’ to ‘Excellent – I know a lot’ were coded as 1–5; Schofield and Forrester-Knauss, 2017). Only one participant did not complete the survey in full, missing one item, which is reported in Table 2. Data from section C of the survey captured whether something was Useful (option 1), Currently used (option 2), More required (option 3), Not useful (option 4) or Unsure (option 5). Data from section C of the survey were then used to determine the relative usefulness of resources (e.g. Useful – Not useful). The relative need was determined by the option ‘more required’. The mean percentage of the relative usefulness and the identified relative need were then used to create a quadrant graph (see Supplemental Appendix B for an example of how these calculations were completed). Quadrants created were as follows: 1 – Identifies resources as being of critical focus (requiring immediate attention – both highly useful and more required); 2 – Identifies resources needing to be maintained (highly useful but more are not required); 3 – Identifies resources requiring addressing at some stage (currently rated as low for usefulness but more are required) and 4 – Identifies resources perceived to be of low priority (currently rated as low for usefulness and more are not required). Higher mean percentages of either relative usefulness or relative need indicate higher levels of usefulness and need, respectively.

Qualitative data generated from open-ended response items were analysed using content analysis. Quotes were organised and sorted using a Microsoft Excel spreadsheet. Qualitative data were coded across three domains (from six extended response questions) relating to sleep. These were (i) the current and possible role of occupational therapists; (ii) facilitators and barriers to practice and (iii) resources currently used or that would be beneficial for practice. Coding was initially conducted by MW and then discussed among the rest of the research team until a consensus on category content was reached (Liamputtong, 2020; Renz et al., 2018).

## Results

A total of 60 online surveys were received. Data from 9 participants were excluded due to incomplete surveys, leaving 51 participant responses included in the data analysis.

### Participant characteristics

Of the 51 participants, the majority were female (92.2%), with an overall average of 15.5 years of experience (SD = 9.5, range 1.5–42). Most participants (76%) reported their highest qualification as a bachelor’s degree in occupational therapy, with just over half of the participants (55%) indicating completion of additional education, training or qualifications specific to palliative care. At the time of the survey, 88% of participants had worked in palliative care for more than 1 year. The range of experience of working as an occupational therapist in palliative care ranged from 6 months to 24 years (*M* = 7.6 years, SD = 6.1). Participants reported working across a range of practice settings, with nearly half providing service in a hospital setting (*n* = 32, 42%). A summary of participant characteristics is provided in Table 1.

**Table 1.** Summary of participant characteristics, *N* = 51.

Characteristic	Respondents (*n*)	Percentage (%)
State or territory of residence		
Australian Capital Territory	2	3.92
New South Wales	13	25.49
Northern Territory	1	1.96
Queensland	7	13.73
South Australia	2	3.92
Tasmania	3	5.88
Victoria	19	37.25
Western Australia	4	7.84
Highest qualification		
Bachelor degree	39	76.47
Masters	12	23.53
Completion of additional education/training/qualification in palliative care		
Yes	28	54.90
No	23	45.10
Years of experience as an occupational therapist working in palliative care		
Less than 1 year	6	11.76
One or more years	45	88.24
Employment setting		
Home care	27	35.06
Hospital care	32	41.56
Hospice care	8	10.39
Residential aged care	2	2.60
Other^a^	8	10.39

a Participants who reported providing palliative care in an employment setting different from the settings listed (such as private practice, including through the National Disability Insurance Scheme).

### Quantitative results

#### Occupational therapy role

The majority of participants (*n* = 45, 88.2%) indicated that sleep is part of the occupational therapy role within palliative care. A small number of participants (*n* = 6, 12%) noted they were unsure or did not perceive sleep as within the role of occupational therapy in palliative care. Few perceived having good or excellent knowledge of sleep physiology (*n* = 9, 25.87%), sleep disorders (*n* = 6, 11.77%) or sleep latency (*n* = 5, 9.80%). Additional data regarding perceived knowledge of occupational therapists in relation to sleep are outlined in Table 2.

**Table 2.** Participants perceived knowledge of sleep, *N* = 51.

Knowledge area	No/poor knowledge		Fair knowledge		Good/excellent knowledge	
	*n*	%	*n*	%	*n*	%
Sleep physiology	20	27.97	22	46.15	9	25.87
Sleep disorders	21	41.18	24	47.06	6	11.76
Sleep hygiene	4	7.84	15	29.41	32	62.75
Sleep latency	28	54.90	18	35.29	5	9.80
Sleep duration expected by age	15	29.41	26	50.98	10	19.61
Sleep–wake cycles	4	7.84	33	64.71	14	27.45
Influence of pain on sleep	6	11.76	16	31.37	29	56.86
Influence of fatigue on sleep	7	13.73	15	29.41	29	56.86
Influence of balance and/or strength disturbance on sleep	25	49.02	20	39.22	6	11.76
Influence of skin integrity issues on sleep	11	21.57	17	33.33	23	45.10
Influence of sensory system disturbances on sleep (e.g. auditory and visual systems)^a^	14	28.00	19	38.00	17	34.00
Influence of breathlessness on sleep	3	5.88	16	31.37	32	62.75
Influence of gastrointestinal disturbances on sleep	15	29.41	16	31.37	20	39.22
Influence of psychological/emotional functioning on sleep (e.g. existential worries)	3	5.88	19	37.25	29	56.86

a Item received 50 participant responses.

When responding to the Yes/No questions: ‘Do you ask about sleep in your initial assessment with clients in palliative care?’ most therapists (*n* = 40, 78%) reported doing so. However, when rating their knowledge about sleep assessments, only 3 (5.88%) study participants had good or excellent knowledge, while less than one-quarter (*n* = 12, 23.53%) of participants reported good or excellent knowledge of sleep interventions. Table 3 outlines additional data regarding perceived knowledge of occupational therapists in relation to sleep assessment and intervention.

**Table 3.** Perceived knowledge of sleep assessment and intervention, *N* = 51.

Knowledge area	No/poor knowledge		Fair knowledge		Good/excellent knowledge	
	*n*	%	*n*	%	*n*	%
Sleep assessments	28	54.90	20	39.22	3	5.88
Sleep interventions	13	25.49	26	50.98	12	23.53
Evidence-based bed positioning	13	25.49	23	45.10	15	29.41
Environmental factors (e.g. noise, temperature, lighting)	2	3.92	18	35.29	31	60.78
Bedding management (e.g. comfort)	3	5.88	17	33.33	31	60.78
Nocturnal toileting safety	5	9.80	11	21.57	35	68.63
Carer education on sleep misconceptions and expectations	12	23.53	22	43.14	17	33.33
Addressing secondary conditions that may decrease sleep quality	14	27.45	21	41.18	16	31.37
Daytime activity programmes	12	23.53	24	47.06	15	29.41
Cognitive-behavioural therapy	20	39.22	22	43.14	9	17.65
Relaxation/meditation techniques for sleep	4	7.84	19	37.25	28	54.90
Progressive muscle relaxation	9	17.65	15	29.41	27	52.94
Mindfulness	4	7.84	16	31.37	31	60.78
Biofeedback	24	47.06	23	45.10	4	7.84

#### Resources identified as useful

**Figure 1.**
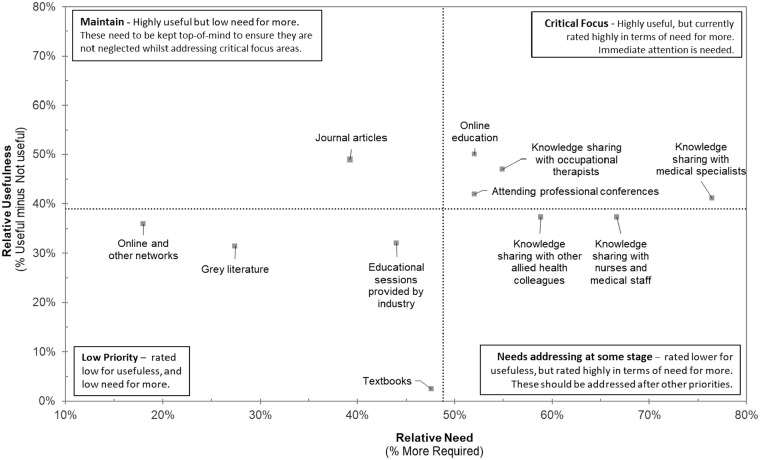
Resources identified as necessary.

Participants were asked to indicate from 11 resources their perceptions regarding usefulness and need. Figure 1 presents the quadrant graph that identifies the actions and resources required to best support occupational therapists’ practice in addressing sleep disturbances for clients in palliative care. Resources in the top right quadrant were identified as being of critical focus (requiring immediate attention). Resources in the top left quadrant need to be maintained, those in the bottom right quadrant require addressing at some stage, and those resources in the bottom left quadrant were perceived to be of low priority.

### Qualitative results

The qualitative data were categorised under the following headings: Occupational therapy role, Facilitators and barriers to practice, and Occupational therapy resources for sleep. Throughout the findings, the terms client and patient are used in relation to the individual receiving occupational therapy, as respondent occupational therapists used these terms interchangeably depending on their service setting.

#### Occupational therapy role

##### Current role

Forty-one participants commented on their role in addressing sleep in palliative care. Two key domains were highlighted: assessment and intervention. Participants noted occupational therapy assessment of sleep quality in palliative care was often limited and addressed informally, with P46 stating ‘assessment of sleep quality [is only] through discussion and identifying possible barriers to quality sleep’ and P42 noting they used ‘subjective assessments only’ to explore an individual’s sleep. Some participants described assessing environmental factors that may influence their client’s sleep: ‘assessment of positioning; environment and symptoms’ (P38); ‘home assessment’ (P24) and ‘their equipment (like beds, mattresses, sleep positioning systems); their supports (like parents/carers); sleep safety (like Sudeen infant Death Syndrome (SIDS), oxygen management); their sleep routines and managing medical needs overnight (like enteral feeds)’ (P33).

Participants highlighted intervention as an additional component of the occupational therapy role in sleep management in palliative care. Interventions listed included the following: equipment prescription; symptom management; education; sleep hygiene; addressing additional environmental factors and liaising with relevant stakeholders. Equipment prescription was reported as a major component of their role: ‘Equipment prescription i.e. pressure relieving mattresses, electric bed, bed wedges, bed rail, and stick’ (P16). Managing symptoms that indirectly influence sleep was also reported by participants: ‘Handover for symptom management. Treatment approaches aim at assisting to manage causative factors of impaired sleep – stress, discomfort, pain, urination’. Education was also a predominant sleep management intervention with clients: ‘Verbal and written education on impacts of sleep in palliative care and particularly cancer care and strategies to assist with same’ (P49); and ‘Education on positioning, trial of positioning aids, education re sleep hygiene and patterns, informal education re mindfulness and breathing’ (P23).

Interventions described less often involved aspects of sleep hygiene: ‘Interventions may be a discussion around sleep hygiene/routine’ (P47) and ‘basic strategies for patient to trial to improve sleep hygiene; provide Sleep Hygiene handout’ (P22). Liaising with relevant stakeholders: ‘discussion and liaison with patient/family/caregivers/nursing staff/medical staff re: use of medications to assist with sleep and potential contraindications’ (P15) and ‘linking in with other allied health or medical as required’ (P48) were also mentioned.

Some participants reported having minimal or no role in sleep management as an occupational therapist in palliative care: ‘Basic, brief sleep assessments and intervention related to sleep on the ward and if relevant home, if we even assess sleep at all’ (P1) and ‘My role currently does not entail a lot to address sleep issues in palliative care clients’ (P17).

##### Possible role

Participants felt there was an opportunity to place greater emphasis on considering sleep disturbances within their palliative care role: ‘More focus on the question of sleep rather than it being incidental to other areas already being addressed’ (P15); ‘To have sleep given equal weighting with other symptoms such as nausea or pain’ (P58). Participants identified potential for further comprehensive sleep assessments: ‘Taking more time to assess and to evaluate someone’s sleep in the palliative phase’ (P23); ‘More in-depth assessment on sleep quality and cycles’ (P27).

The potential for further intervention, such as enhanced liaison with stakeholders, was strongly reported. Comments included the following: ‘referring to the correct pathways’ (P4), for example, other members of the multidisciplinary team. Participants described ‘Liaising with medical/nursing staff about the client’s sleep issue so that medical intervention can be addressed as appropriate – it requires a multidisciplinary approach. Liaise with other allied health staff as needed’ (P46).

#### Facilitators and barriers to practice

##### Facilitators

Thirty-six participants described two key factors facilitating their ability to address sleep as part of their current role: adequately resourced workplaces and the scope of occupational therapy practice. Being adequately resourced enabled therapists to have sufficient time to assess and address patients’/clients’ sleep: ‘time to include sleep in a thorough initial assessment’ (P26) and ‘Time to address and provide intervention and follow up’ (P14). Having accessible information and resources enabled them to appropriately address sleep issues: ‘able to access limited equipment’ (P36) and ‘access to appropriate interventions to address specific sleep issues’ were required (P40). The importance of input and support from other members of the multidisciplinary team, and from senior occupational therapists, further facilitated participants to address sleep issues among their patients: ‘ability to work well with other disciplines to explore options, good educators’ (P15), ‘networking with other palliative care OTs’ (P16).

##### Barriers

Forty-four participants identified barriers to addressing sleep issues within their palliative care role. Three key barriers were identified: service constraints/limitations; limited experience, knowledge, and skills; and poor understanding of the occupational therapy role by the multidisciplinary team.

Participants indicated that workload constraints such as ‘caseload demands’ (P32) limited their ability to address sleep disturbances and often meant these were not prioritised in service delivery. For example, participant 32 noted there was a ‘lack of time for assessment, intervention & documentation’ (P32). Limited resources were identified as an additional challenge. Participant 34 stated there was ‘limited access to pall care specific resources’ (P34); while participant 40 said there was a ‘lack of access to intervention’ (P40). Participants identified that a lack of staff limited their ability to address sleep issues. For example, ‘staffing allocation resulting in minimal availability for input and follow up’ (P45).

Participants recognised their own limited experience, knowledge and skills as a challenge to their ability to address sleep disturbances among their clients/patients. Participants referred to ‘personal lack of knowledge – minimal experiences’ (P20); ‘lack of confidence/experience in this area’ (P54) and ‘lack of in-depth knowledge about sleep disorders and interventions’ (P46).

Some participants noted that limited understanding by the multidisciplinary team of their role was a barrier to the scope of practice in sleep management, such as one participant who stated that there was a ‘lack of understanding of OT scope’ (P3). This limited understanding was recognised as preventing appropriate and timely referrals for occupational therapy services, impacting the ability of occupational therapists to adequately address sleep issues. Examples provided were as follows: ‘lack of understanding of MDT in OT role in this area – referrals not received’ (P54), and ‘receiving referrals early enough so patients have time to put strategies into place’ (P13).

#### Occupational therapy resources for sleep

##### Resources currently used in occupational therapy practice

Twenty-four participants provided information regarding resources they were currently using to address sleep issues in palliative care. Five main types of resources were identified: sleep hygiene handouts; relaxation and mindfulness techniques; information from websites; personally developed materials and other resources. Participant 49 said they used ‘patient handouts on the impact of sleep and strategies to assist’. A small number of participants outlined using personally developed handouts and materials as part of their role in sleep management. Relaxation and mindfulness techniques were used to address sleep management with clients. Examples provided included ‘relaxation/progressive muscle relaxation CD’ (P26), ‘mindfulness scripts and CD’ (P36) and ‘a kit that focuses heavily around mindfulness approaches to improving sleep’ (P53). Information from websites was identified as the most commonly used resource, with one participant noting they ‘mainly used information from other institutional websites regarding sleep hygiene practices’ (P46). Occupational therapists also identified using their own experience and knowledge to support the sleep management of their patients/clients. Specifically, participants reported ‘experience across other clinical areas’ (P24), ‘pressure care, comfort and positioning’ (P7) and ‘energy conservation and strategies to assist with sleeping’ (P4).

##### Resources that would enhance occupational therapy practice

Nineteen participants identified resources that could potentially be useful to them to support their occupational therapy practice. The provision of more professional development opportunities would enhance clinician knowledge and better position occupational therapists to address sleep in palliative care settings. Examples provided included the following: ‘Better access to formal edu[cation] and PD in this area’ (P54); ‘workshops on different topics/areas of addressing sleep in palliative care, and how OT can play a role’ (P16); Evidence-based guidelines were identified as important: ‘evidence-based guidelines’ (P42); ‘I would like to have more of a “toolkit” for sleep issue interventions’ (P20); ‘More data and evidence to support the interventions’ (P53).

## Discussion

This study has provided an overview of the occupational therapy role in Australian palliative care in relation to sleep. Sleep was acknowledged as important and within the scope of practice by occupational therapists in palliative care; however, several implementation barriers were identified.

Assessment of sleep was identified as a key component of the occupational therapy role; however, this was often incidental and completed through brief, subjective methods, with participants reporting limited knowledge of specific sleep assessments. This aligns with previous research in palliative care settings, which identified that sleep disturbances are often inadequately addressed by health professionals with palliative care and cancer patients (Laugsand et al., 2011; Nzwalo et al., 2020). Lack of standardised routine assessments of sleep may act as a barrier for occupational therapists providing suitable interventions and further contribute to sleep issues being under-recognised and under-addressed in palliative care and occupational therapy practice (Faulkner and Mairs, 2015; Fung et al., 2013). Sleep disturbances can profoundly influence individual function and quality of life and detrimentally affect cognitive function, physical comfort and emotional status at the end of life (Klingman and Boyce, 2018). If not adequately identified and addressed by health professionals such as occupational therapists, sleep disturbances may further contribute to reduced engagement in social relationships and reduced participation in meaningful occupation, decreasing quality of life and well-being at the end of life.

Occupational therapists in this study reported using equipment prescription, education, sleep hygiene, addressing additional environmental factors and liaising with relevant stakeholders as the main sleep interventions within their role. This is consistent with previous research with other populations that identified the importance of environmental adaptations, such as noise and lighting, as key intervention strategies to address sleep issues (Faulkner and Mairs, 2015). In addition, occupational therapists can implement daytime activity programmes and interventions to indirectly aid sleep, develop occupational balance and restructure activity to promote engagement in meaningful occupation in their role in sleep management (Ho and Siu, 2018). Yet, therapists in our study acknowledged that they had limited knowledge of activity programmes.

Participants in this study identified service-related factors such as time constraints, workload restrictions and limited resources as the predominant barriers to sleep intervention. These are similar to the barriers identified in an Australian cross-sectional survey of 11 occupational therapists conducted by Wallis et al. (2022), which highlighted the lack of time and limited funding as impacting occupational therapists’ ability to provide adequate care for young people in palliative care. Similarly, a survey of 108 palliative care nurses in Central New York State identified institutional constraints, including poor institutional support and intervention costs, as key barriers to the provision of non-pharmacological sleep-promoting interventions for nurses in palliative care (Capezuti, 2021). High caseload demands in conjunction with limited time available may require occupational therapists to prioritise immediate safety and risk management issues, such as pressure care management and equipment provision. This may result in less time to focus on other interventions such as activity programmes and morning and evening routines. In addition to aiding sleep quality, such interventions are also of great importance for individuals at the end of their lives.

The present study highlights the need for occupational therapists to have access to more resources to enhance their knowledge about, and skills to address, sleep with their palliative care patients. Occupational therapists may benefit from increased professional development opportunities and the development of evidence-based resources and guidelines to support their capacity to effectively address sleep disturbances and enhance client care in palliative care settings. Educational resources provided by governments, palliative care organisations, occupational therapy associations, universities and funding bodies may subsequently be required to enhance the knowledge, confidence and skills of occupational therapists in this practice area to facilitate high-quality, holistic client-centred practice and augment client outcomes (Faulkner and Mairs, 2015).

Participants in our study felt further limited in effectively assessing and addressing sleep by limited practice recommendations and guidelines. Most participants highlighted the importance of addressing sleep issues; however, at times were unsure how best to do so due to a lack of clinical practice guidelines. Further clarification is needed regarding practice recommendations and guidelines to better equip occupational therapists to incorporate sleep management in daily practice (Faulkner and Mairs, 2015).

Unless institutional barriers such as lack of funding and time restrictions allocated to treat sleep disturbances are adequately addressed, then it is likely that sleep issues will continue to receive suboptimal service provision in clinical practice (Green and Brown, 2015; McVeigh et al., 2022). Funding bodies and employers need to recognise the importance of addressing sleep issues in palliative care, its practice implications, and how occupational therapy is positioned to effectively address sleep issues to enhance engagement and quality of life in palliative care (Green and Brown, 2015; McVeigh et al., 2022).

### Limitations

This study has some limitations to acknowledge, which could reduce the generalisability of the findings. The survey used was a non-validated survey; therefore, there is potential for measurement error in accurately measuring the intended concept of sleep. Self-selection bias may be a factor as non-probability sampling methods were employed and participants self-selected; therefore, participants interested in sleep may have been more inclined to undertake the survey. However, 12% of participants did report being unsure or did not perceive sleep as within the role of occupational therapy in palliative care. Furthermore, elaboration of the types of populations with whom the participants worked in palliative care (e.g. ages, diagnoses) could have added clarity to the sample description. Although the survey received responses from all states and territories across Australia, higher response rates were received from New South Wales and Victoria.

## Conclusion

This study has provided important insights into the role, scope and implementation of sleep management interventions among Australian occupational therapists within palliative care, and the needs that therapists have to support this area of practice. Occupational therapists perceived sleep to be within their scope of practice in palliative care and have a unique skillset to offer to support sleep health. However, they lack certainty of knowledge in relation to sleep assessment and interventions. To better support their patients’ sleep-related needs in palliative care, the occupational therapy profession would benefit from the development and implementation of resources to support practice, including increased professional development opportunities, training on implementing activity programmes and evidence-based guidelines.

Several service-related barriers to best practice were identified, including time constraints and limited funding. In addition, there was a lack of understanding of the scope of occupational therapists by other team members. By raising awareness among palliative care service management and other professionals regarding the important role of occupational therapists in this domain, there may be more referrals to address sleep issues, in addition to greater resources allocated to address this critical need.

**Key findings**

- Professional development, evidence-based resources and clinical practice guidelines are needed to support occupational therapists’ confidence in addressing sleep issues in palliative care.
- Organisational barriers such as workload constraints and lack of time need to be addressed to enable occupational therapists to better support their palliative patients’ sleep-related needs.
- Heightened awareness among palliative care service management and other professionals regarding the important role of occupational therapists in this domain is needed.

**What the study has added**

The study provides new information regarding the role of occupational therapists in addressing sleep issues in palliative care and identifies the facilitators and barriers to implementing sleep-related interventions.

## Supplemental Material

sj-docx-1-bjo-10.1177_03080226251352648 – Supplemental material for Occupational therapists’ role in sleep management in palliative care: A cross-sectional survey Supplemental material, sj-docx-1-bjo-10.1177_03080226251352648 for Occupational therapists’ role in sleep management in palliative care: A cross-sectional survey by Madeleine Webster, Linda Barclay, Dhwani Parikh and Aislinn Lalor in British Journal of Occupational Therapy

sj-docx-2-bjo-10.1177_03080226251352648 – Supplemental material for Occupational therapists’ role in sleep management in palliative care: A cross-sectional survey Supplemental material, sj-docx-2-bjo-10.1177_03080226251352648 for Occupational therapists’ role in sleep management in palliative care: A cross-sectional survey by Madeleine Webster, Linda Barclay, Dhwani Parikh and Aislinn Lalor in British Journal of Occupational Therapy
