# Supplementary material for: Occupational therapists’ role in sleep management in palliative care: A cross-sectional survey
Source: Br J Occup Ther. 2025 Jul 21;88(12):746–55. doi: 10.1177/03080226251352648 (PMC12623621; doi:10.1177/03080226251352648)
Supplement: sj-docx-1-bjo-10.1177_03080226251352648 – Supplemental material for Occupational therapists’ role in sleep management in palliative care: A cross-sectional survey [file sj-docx-1-bjo-10.1177_03080226251352648.docx]

**Appendix A - Complete Survey**

Section A: Demographics
The first section of this survey asks you some questions about yourself. Please select the box or fill in the answer that best describes you.

Q1 What is your gender?

o Woman (1)

o Man (2)

o My gender isn't listed - I identify as: (3) ____________________

o I prefer not to say (4)

Q2 What is your age in years? ______________

Q3 Which state or territory of Australia are you located in?

o Australian Capital Territory (1)

o New South Wales (2)

o Northern Territory (3)

o Queensland (4)

o South Australia (5)

o Tasmania (6)

o Victoria (7)

o Western Australia (8)

Q4 What is your highest level of occupational therapy education completed?

o Bachelor’s degree (1)

o Master’s degree (2)

o Doctoral degree (3)

o Other (please state): (4) __________________________________

Q5 How many years have you been working (or worked) in the field of occupational therapy?

o Less than one year (1)

o More than one year (please specify the number of years) (2) ________________

Q6 How many years have you been working (or worked) in the field of palliative care in occupational therapy?

o Less than one year (1)

o More than one year (please specify the number of years) (2) ________________

Q7 Where do/did you provide palliative care as an occupational therapist? (Select all that apply)

▢ Home care (1)

▢ Hospital care (2)

▢ Hospice care (3)

▢ Residential aged care (4)

▢ Other (please state): (5) _____________________

Q8 Have you completed additional education/training/qualification specific to palliative care to complement your occupational therapy education?

o Yes (please state): (1) ______________________

o No (2)

Section B: Occupational Therapy Role in Sleep in Palliative Care

This section aims to explore your understanding of sleep and your perception of the role of occupational therapy regarding sleep in palliative care. Please answer the following questions according to the instructions provided.

Q9 Do you believe sleep is part of the role of occupational therapy working in palliative care?

o Yes (1)

o No (2)

o Unsure (3)

Q10 Do you ask about sleep in your initial assessment with clients in palliative care?

o Yes (1)

o No (2)

Q11 How would you rate your knowledge about each of the following areas regarding sleep? Please select the box that best describes you.

|  | I have no knowledge (1) | Poor - I know very little (2) | Fair - I know a bit (3) | Good - I know quite a bit (4) | Excellent - I know a lot (5) |
| --- | --- | --- | --- | --- | --- |
| Sleep physiology (1) | o | o | o | o | o |
| Sleep disorders (2) | o | o | o | o | o |
| Sleep hygiene (3) | o | o | o | o | o |
| Sleep latency (4) | o | o | o | o | o |
| Sleep duration expected by age (5) | o | o | o | o | o |
| Sleep-wake cycles (6) | o | o | o | o | o |
| Influence of pain on sleep (7) | o | o | o | o | o |
| Influence of fatigue on sleep (8) | o | o | o | o | o |

Q12 How would you rate your knowledge about each of the following areas regarding sleep? Please select the box that best describes you.

|  | I have no knowledge (1) | Poor - I know very little (2) | Fair - I know a bit (3) | Good - I know quite a bit (4) | Excellent - I know a lot (5) |
| --- | --- | --- | --- | --- | --- |
| Influence of balance and/or strength disturbance on sleep (9) | o | o | o | o | o |
| Influence of skin integrity issues on sleep (10) | o | o | o | o | o |
| Influence of sensory system disturbances on sleep (e.g., auditory, and visual systems) (11) | o | o | o | o | o |
| Influence of breathlessness on sleep (12) | o | o | o | o | o |
| Influence of gastrointestinal disturbances on sleep (13) | o | o | o | o | o |
| Influence of psychological/emotional functioning on sleep (e.g., existential worries) (14) | o | o | o | o | o |

Q13 How would you rate your knowledge about each of the following areas regarding sleep? Please select the box that best describes you.

|  | I have no knowledge (1) | Poor - I know very little (2) | Fair - I know a bit (3) | Good - I know quite a bit (4) | Excellent - I know a lot (5) |
| --- | --- | --- | --- | --- | --- |
| Sleep assessments (1) | o | o | o | o | o |
| Sleep interventions (2) | o | o | o | o | o |
| Evidence-based bed positioning (3) | o | o | o | o | o |
| Environmental factors (e.g., noise, temperature, lighting) (4) | o | o | o | o | o |
| Bedding management (e.g., comfort) (5) | o | o | o | o | o |
| Nocturnal toileting safety (6) | o | o | o | o | o |
| Carer education on sleep misconceptions and expectations (7) | o | o | o | o | o |
| Addressing secondary conditions that may precipitate decreased sleep quality (e.g., arthritis) (8) | o | o | o | o | o |

Q14 How would you rate your knowledge about each of the following areas regarding sleep? Please select the box that best describes you.

|  | I have no knowledge (1) | Poor - I know very little (2) | Fair - I know a bit (3) | Good - I know quite a bit (4) | Excellent - I know a lot (5) |
| --- | --- | --- | --- | --- | --- |
| Daytime activity programs (9) | o | o | o | o | o |
| Cognitive- Behavioural Therapy (10) | o | o | o | o | o |
| Relaxation /meditation techniques for sleep (11) | o | o | o | o | o |
| Progressive muscle relaxation (12) | o | o | o | o | o |
| Mindfulness (13) | o | o | o | o | o |
| Biofeedback (14) | o | o | o | o | o |
| Stimulus control therapy (15) | o | o | o | o | o |
| Sleep restriction therapy (16) | o | o | o | o | o |
| Sleep aids (17) | o | o | o | o | o |

Section C: Resources identified as useful for occupational therapy practice

This section aims to explore the resources that you identify as: 1) useful; 2) are currently using and 3) require more of to aid occupational therapists when conducting an assessment or providing an intervention for sleep management for people receiving palliative care. Please also indicate if you have found a resource that is NOT useful when conducting an assessment or making a recommendation for sleep management for people receiving palliative care.

Q15 Please select all that apply.

|  | Useful (1) | Currently using (2) | More required (3) | Not useful (4) | Unsure (5) |
| --- | --- | --- | --- | --- | --- |
| Knowledge sharing with occupational therapists (1) | ▢ | ▢ | ▢ | ▢ | ▢ |
| Knowledge sharing with other allied health colleagues (2) | ▢ | ▢ | ▢ | ▢ | ▢ |
| Knowledge sharing with nurses and medical staff (3) | ▢ | ▢ | ▢ | ▢ | ▢ |
| Knowledge sharing with medical specialists (4) | ▢ | ▢ | ▢ | ▢ | ▢ |

Q16 Please select all that apply.

|  | Useful (1) | Currently using (2) | More required (3) | Not useful (4) | Unsure (5) |
| --- | --- | --- | --- | --- | --- |
| Textbooks (1) | ▢ | ▢ | ▢ | ▢ | ▢ |
| Journal articles (2) | ▢ | ▢ | ▢ | ▢ | ▢ |
| Grey literature (e.g. factsheets, reports, proceedings) (3) | ▢ | ▢ | ▢ | ▢ | ▢ |
| Attending professional conferences (4) | ▢ | ▢ | ▢ | ▢ | ▢ |

Q17 Please select all that apply.

|  | Useful (1) | Currently using (2) | More required (3) | Not useful (4) | Unsure (5) |
| --- | --- | --- | --- | --- | --- |
| Online education (e.g. webinars, podcasts, blogs) (1) | ▢ | ▢ | ▢ | ▢ | ▢ |
| Online and other networks (e.g. Facebook groups, special interest groups, support groups) (2) | ▢ | ▢ | ▢ | ▢ | ▢ |
| Educational sessions provided by industry (3) | ▢ | ▢ | ▢ | ▢ | ▢ |

Q18 Please provide details of any other resources that you currently use for sleep management in palliative care:

Q19 Please provide details of any other resources that you think would be useful for sleep management in palliative care:

Section D: Extended Response

The following questions aim to gather a more comprehensive understanding of the role of occupational therapists' in sleep management with clients in palliative care. Please consider your responses to each question carefully and give as much detail as necessary in your answers.

Q20 What does (or did) your role entail in addressing sleep issues for clients receiving palliative care? (E.g., this may relate to assessment, intervention, management, etc).

Q21 What would you like (or would have liked) your role to entail in addressing sleep issues for clients receiving palliative care? (E.g., this may relate to assessment, intervention, management, etc).

Q22 What do you think occupational therapists could be doing to address sleep issues for clients in palliative care?

Q23 What are the facilitators to you as an occupational therapist in addressing sleep issues for clients receiving palliative care?

Q24 What are the barriers to you as an occupational therapist in addressing sleep issues for clients receiving palliative care?

Q25 Is there anything else you would like to add that has not already been covered? Please list in the space provided below.
