# Supplementary material for: Occupational therapists’ role in sleep management in palliative care: A cross-sectional survey
Source: Br J Occup Ther. 2025 Jul 21;88(12):746–55. doi: 10.1177/03080226251352648 (PMC12623621; doi:10.1177/03080226251352648)
Supplement: sj-docx-2-bjo-10.1177_03080226251352648 – Supplemental material for Occupational therapists’ role in sleep management in palliative care: A cross-sectional survey [file sj-docx-2-bjo-10.1177_03080226251352648.docx]

**Appendix B: Example calculations for Section C survey data**

Data from Section C of the survey was used to create the Quadrant Figure in the article. Data was (or wasn’t) included as follows:

1. Useful (included)
2. Currently using (not included)
3. More required (e.g. identified need of the resource) (included)
4. Not useful (included)
5. Unsure (not included)

The relative usefulness of resources was determined by Useful (option 1) minus Not useful (option 4). For example, in relation to the resource “Knowledge sharing with occupational therapists”, the raw data is as follows:

| Resource | Useful | Currently using | More required | Not useful | Unsure | Non-  response |
| --- | --- | --- | --- | --- | --- | --- |
| Knowledge sharing with occupational therapists | 24 | 16 | 28 | 0 | 1 | 0 |

Relative usefulness in this instance was calculated by ‘Useful’ minus ‘Not useful’ (e.g. 24-0=24). Relative need was the column ‘More required’ (e.g. 28) – option 3 from above.

The mean percentage of the relative usefulness and the identified need (option 3) were used to create the quadrant graph shown within the body of the paper. The relative usefulness percentage was calculated in Excel as follows:

=SUM((100/51)*24)=47%

where 51 is the sample size (respondents) and 24 is the relative usefulness.

The relative need percentage was calculated in Excel as follows:

=SUM((100/51)*28)=55%

where 51 again is the sample size (respondents) and 28 is the relative need.

In the quadrant graph, the Relative Need is plotted on the x-axis and the Relative Usefulness is plotted on the y-axis. The quadrants are created through the intersection of the average of the Relative Need of all the resources listed in Section C (e.g. 49%) by the Relative Usefulness of all the resources listed (e.g. 39%).
